# Mevalonate kinase somatic mosaicism and bigenic genotypes may explain heterogeneity in mevalonate kinase deficiency

**DOI:** 10.1186/1546-0096-13-S1-P48

**Published:** 2015-09-28

**Authors:** Y Shinar, PJ Hashkes, R Cohen, A Kessel, I Tirosh, S Padeh, J Arostegui, A Livneh

**Affiliations:** 1 Sheba Medical Center, Ramat Gan, Israel; 2 Shaare Zedek Medical Center, Jerusalem, Israel; 3 Bnei Zion Medical Center, Haifah, Israel; 4 Hospital Clinic, Barcelona, Spain

## Question

The diagnosis of mevalonate kinase deficiency is often delayed, due to the rarity and phenotypic heterogeneity of the disease. We evaluated the autoinflammatory genetic makeup of 4 referrals suspected to have MKD.

## Methods

Exons 2-11 of the mevalonate kinase gene, exon 10 of MEFV, exons 2-4 of TNFRSF1A and exon 3 of NLRP3 were Sanger sequenced in referrals with an autoinflammaory disease. Targeted massive parallel sequencing by the PGM Ion Torrent platform was performed on one peripheral blood DNA sample.

## Results

Case 1 A pediatric case with splenomegaly, cervical lymphoadenopathy, failure to thrive and anemia, was found to have two pathogenic MVK variants, p.V250I and p.L315G*51, and the Q705K variant on the NLRP3 gene, considered a functional polymorphism. Typical symptoms and a high level of urinal mevalonic acid allowed closure on MKD diagnosis this case.

Case 2 A pediatric case with recurrent and vaccination triggered attacks of high fever, sore throat, cervical lymphadenopathy and abdominal pain since infancy was a carrier of the MEFV p.V726A variant. The patient was diagnosed with PFAPA and treated with steroids. In the last, 10 days long attack the patient developed arthritis, a maculopapular rash and high blood pressure. MKD genetic testing revealed two pathogenic MVK variants, p.V377I and p.G202R, confirming the diagnosis of MKD.

Case 3 A patient with adult onset Still's disease symptoms including fever attacks, arthralgia, urticaria, pericarditis, and partial response to NSAIDs was shown to have a rare MVK variant, p.R121W, and the NLRP3 p.Q705K functional polymorphism. The diagnosis is yet unresolved.

Case 4 An adult patient developed high fever and abdominal pain lasting 2-3 and HLA-B51 positive aphthous stomatitis. We identified somatic mosaicism (22%) for a novel, p.Ala161Thr MVK variant. The patient's attacks resolved with anti TNF treatment and the current diagnosis is Behcet's disease.

## Conclusion

Four referrals suspected to have MKD had in common autoinflammatory variants in two autoinflammatory genes. The MVK genotypes of two pediatric patients met the recessive mode of MKD inheritance. By contrast, two late onset patients had somatic/heterozygous MVK variants of unknown clinical significance but may be placed at the milder clinical range of MKD**.**

## Consent to publish

Written informed consent for publication of their clinical details was obtained from the patient/parent/guardian/relative of the patient.

